# Major lower extremity amputation: a contemporary analysis from an academic tertiary referral centre in a developing community

**DOI:** 10.1186/s12893-019-0637-y

**Published:** 2019-11-13

**Authors:** Qusai Aljarrah, Mohammed Z. Allouh, Sohail Bakkar, Abdelwahab Aleshawi, Hasan Obeidat, Emad Hijazi, Nabil Al-Zoubi, Heba Alalem, Tagleb Mazahreh

**Affiliations:** 1 Department of Surgery, Faculty of Medicine, Jordan University of Science and Technology, P.O. Box 3030, Irbid, 22110 Jordan; 2 Department of Anatomy, Faculty of Medicine, Jordan University of Science and Technology, P.O. Box 3030, Irbid, 22110 Jordan; 3 Department of Anatomy, College of Medicine & Health Sciences, United Arab Emirates University, Al Ain, 17666 UAE; 4 Department of Surgery, Faculty of Medicine, The Hashemite University, P.O. Box 330127, Zarqa, 13133 Jordan; 5 School of Medicine, Faculty of Medicine, Jordan University of Science and Technology, P.O. Box 3030, Irbid, 22110 Jordan

**Keywords:** Amputation, Lower extremity, Peripheral arterial disease, Diabetic foot

## Abstract

**Background:**

We aimed to explore the surgical outcomes of major lower extremity amputation (MLEA) and influencing factors at an academic tertiary referral centre in north Jordan, optimistically providing a platform for future health care policies and initiatives to improve the outcomes of MLEA in Jordan.

**Methods:**

Clinical records of patients who had undergone MLEA between January 2012 and December 2017 were identified and retrospectively reviewed. *International Classification of Diseases* codes were used to identify the study cohort from a prospectively maintained computerised database. We included adult patients of both genders who underwent amputations for ischemic lower limb (acute and chronic) and diabetic foot syndrome (DFS). We excluded patients for whom MLEA surgery was performed for other indications (trauma and tumors). Outcomes of interest included patient demographics and comorbidities, type of amputation and indications, length of hospital stay (LOS), the need for revision surgery (ipsilateral conversion to a higher level of amputation), and cumulative mortality rate at 1 year. The impact of the operating surgeon’s specialty (vascular vs. non-vascular surgeon) on outcomes was evaluated.

**Results:**

The study cohort comprised 140 patients who underwent MLEA (110 below-knee amputations [BKA] and 30 above-knee amputations [AKA]; ratio: 3:1; 86 men; 54 women; mean age, 62.9 ± 1.1 years). Comorbidities included diabetes, hypertension, dyslipidaemia, ischaemic heart disease, congestive heart failure, chronic kidney disease, stroke, and Buerger disease. The only associated comorbidity was chronic kidney disease, which was more prevalent among BKA patients (*p* = 0.047). Indications for MLEA included DFS, and lower limb ischaemia. Acute limb ischaemia was more likely to be an indication for AKA (*p* = 0.006). LOS was considerably longer for AKA (*p* = 0.035). The cumulative mortality rate at 1 year was 30.7%. Revision surgery rates and LOS improved significantly with increased rate of vascular surgeon-led MLEA.

**Conclusions:**

In developing countries, the adverse impact of MLEA is increased because of limited resources and increased prevalence of diabetes-related foot complications. Vascular surgeon-led MLEA is associated with decreased revision rates, LOS and possibly improved outcomes, particularly when it is performed for vascular insufficiency. It is important to formulate national health care policies to improve patient outcomes in these countries.

## Background

Major lower extremity amputation (MLEA) is a life-changing procedure that results in significant morbidity and mortality worldwide. The adverse social implications of and effects on the capacity to work, quality of life, and self-image are devastating. The reported incidence of non-traumatic MLEA is between 12 and 50 per 100,000 individuals per year [1]. In 2010, *Salman* et al. reported an MLEA incidence of 19.3/100,000 patients per year in Jordan [2]. The magnitude of this problem is anticipated to increase with the aging global population and increased prevalence of diabetes [3–5].

Although these countries are acquiring modern trends of treating peripheral vascular disease, reports of the outcomes of MLEA in Middle Eastern and North African countries are sparse [6]. The true impact of MLEA in developing countries needs to be determined in the context of the lack of integral rehabilitation facilities and assisted living services, as well as limited financial resources. The purpose of this study was to provide a comprehensive analysis of MLEA surgical outcomes in Jordan to formulate policies and undertake initiatives that could help improve outcomes for this group of patients.

This study comprehensively analysed contemporary MLEA practices, patterns, and annual trends in Jordan. Amputations performed by vascular surgeons are associated with reduced revision rates and LOS, and presumably, reduced costs. Predictors of 1-year mortality include female gender, older age, hypertension, stroke, CKD, and low serum albumin levels.

MLEA is associated with high morbidity and mortality. In fact, it remains the only procedure on record in surgical history with a 300% mortality rate. No other procedure has achieved this record [7]. Furthermore, there is always a sense of failure associated with amputation because we have failed to salvage a limb; however, this does not change the fact that it must be performed.

The literature contains disparities regarding the morbidity and mortality trends of patients who have undergone MLEA. Despite the recent advances in vascular and endovascular therapy, amputation trends have not changed significantly over the past two decades [8–11]. In a recent systematic review, *Narres* et al. explored the global MLEA trends in diabetic and non-diabetic patients [5]. They found great variations in the international trends despite the heterogeneity in the reported literature. Consequently, they recommended a unified structure for future studies so that trends and relative risks of MLEA could be analysed more precisely [5].

Undoubtedly, patients requiring MLEA represent a group of seriously unwell patients. They impose a great impact on the health care system, especially when more than 150,000 MLEA procedures are performed annually worldwide [12].

## Materials and methods

After obtaining approval from the Institutional Review Board (IRB), we examined our institution’s medical record database and retrospectively identified patients who had undergone MLEA between January 2012 and December 2017 using *International Classification of Diseases* (9th edition) codes 84.1, 84.17, and 84.3. Electronic medical records were retrieved and standard demographic information, comorbidities, level of amputation, length of hospitalisation, ipsilateral revision rate, subsequent contralateral amputation, and 1-year mortality data were obtained.

Indications for MLEA included diabetic foot syndrome (DFS), acute limb ischaemia, and chronic limb ischaemia. The authors excluded traumatic MLEA and amputations performed for other indications. Major amputations included below-knee amputations (BKA) and above-knee amputations (AKA). Revision was defined as requiring ipsilateral AKA after BKA. Patients who underwent BKA that was converted to AKA during the study period were included in the AKA group. Mortality was defined at 1 year after the index MLEA, and it included in-hospital deaths. Patients were diagnosed with chronic kidney disease (CKD) if they met one of the following criteria: 1) a diagnosis of CKD according to hospital records, 2) use of dialysis, 3) a history of kidney transplantation with renal impairment or relapse, or 4) glomerular filtration rate less than 60 mL/min. The last documented laboratory values before the index MLEA were used for the analysis. Serum albumin and HbA1c levels were evaluated as numerical variables, with normal ranges of 3.5 to 5.5 g/dL and 4 to 5.6%, respectively. The length of hospital stay (LOS) was defined as the time from hospital admission to discharge following the amputation.

### Setting

In our institute, all vascular surgeon-led amputations were performed by a consultant vascular surgeon or a postgraduate year 4/5 vascular surgery trainee in the presence of a supervising consultant vascular surgeon. The non-vascular surgeon-led amputation service included consultant general surgeons and post graduate year 4/5 general surgery trainees. All surgeries were performed at a single academic institution, the King Abdullah University Hospital (KAUH), which is affiliated with the Jordan University of Science and Technology.

### Statistical analyses

Data were entered into a spreadsheet. Statistical analyses were performed using appropriate software. *P* < 0.05 indicated a statistically significant difference. The preoperative and postoperative factors related to MLEA were described using the frequency distribution for categorical variables and the mean ± standard error (SE) of the mean for continuous variables. Then, data were examined using Pearson’s χ [2] test of association for categorical variables and Student’s *t*-test and one-way analysis of variance (ANOVA) for continuous variables. If a significant (*P* < 0.05) relationship was found, then a post hoc residual analysis for categorical variables and a Fisher’s least significant difference test for continuous variables were applied to determine the exact significance between groups for each variable.

## Results

### Patient characteristics

The study cohort included 140 patients who underwent MLEA at KAUH between 2012 and 2017 (110 BKA, 30 AKA) over a 6-year period. Approximately two-thirds of these patients were men (61.4%). The average age of the amputee patients was approximately 63 years (62.1 years for BKA; 66.0 years for AKA). There was a high prevalence of comorbid medical conditions (Table 1). The most common comorbidity was diabetes (89.3%), followed by hypertension (80.3%) and hyperlipidaemia (65.0%). As expected for arteriopathy patients, 46% had ischemic heart disease, 31.6% had congestive heart failure, and 27.7% had cerebrovascular disease. Nearly one-quarter of patients had CKD. Nineteen patients had Buerger disease (14.2%).

**Table 1:** Demographic distribution of major lower limb amputations in northern Jordan between 2012 and 2017

Preoperative and postoperative variables	Number	%
	Mean ± SE	
Sex		
Male	86	61.4
Female	54	38.6
Age (years)	62.9 ± 1.1	
Comorbidities		
Diabetes mellitus	125	89.3
Hypertension	110	80.3
Dyslipidaemia	91	65.0
Ischaemic heart disease	63	46.0
Congestive heart failure	43	31.6
Chronic kidney disease	39	28.5
Stroke	38	27.7
Buerger disease	19	14.2
Indication for amputation		
Diabetic foot syndrome	102	72.9
Chronic limb ischaemia	20	14.3
Acute limb ischaemia	18	12.8
Ulceration	118	84.3
Laboratory values		
Albumin	29.5 ± 0.7	
HbA1c	8.9 ± 0.2	
Level of amputation		
BKA	110	78.6
AKA	30	21.4
Side		
Right	73	52.9
Left	67	47.1
Bilateral amputation	37	26.4
Revision	14	10.0
LOS (mean ± SE, days)	6.8 ± 0.4	
Death	43	30.7

*SE* Standard error, *HbA1c* Haemoglobin A1c, *BKA* Below-knee amputation, *AKA* Above-knee amputation, *LOS* Length of stay

The most common indication for amputation, as defined by the 9th edition of the *International Classification of Diseases*, was DFS (72.9%), followed by lower limb ischaemia (acute and chronic; 27.1%). The preoperative and postoperative characteristics of these patients are provided in Table 1.

### Level of amputation

There were no significant associations between the level of amputation and the age or sex of the patients. In addition, there was no significant association between the level of amputation and different comorbidities, except for CKD. Patients with CKD were significantly more likely to undergo BKA instead of AKA (*P* < 0.05). Regarding the indications for amputation, acute ischaemia was significantly associated with AKA (*P* < 0.05) (Table 2).

**Table 2:** Distribution and analysis of preoperative and postoperative factors associated with major lower limb amputations in northern Jordan according to the level of amputation

Preoperative and postoperative variables	BKA N (% from BKA)	AKA N (% from AKA)	*P*-value
Sex			
Male	67 (60.9)	19 (63.3)	NS
Female	43 (39.1)	11 (36.7)	
Age (mean ± SE, years)	62.1 ± 1.3	66.0 ± 2.2	NS
Comorbidities			
Hypertension	87 (80.6)	23 (79.3)	NS
Diabetes mellitus	98 (89.1)	27 (90.0)	NS
Ischaemic heart disease	49 (45.4)	14 (48.3)	NS
Congestive heart failure	33 (30.8)	10 (34.5)	NS
Stroke	32 (29.6)	6 (20.7)	NS
Chronic kidney disease	35 (32.1)	4 (14.3)^↓^	0.047*
Dyslipidaemia	73 (66.4)	18 (60.0)	NS
Buerger disease	16 (14.8)	3 (11.5)	NS
Indication for amputation			
Acute limb ischaemia	9 (8.2)	9 (30.0)^↑^	0.006*
Diabetic foot syndrome	84 (76.4)^↑^	18 (60.0)	
Chronic limb ischaemia	17 (15.5)	3 (10.0)	
Ulceration	96 (87.3)	22 (73.3)	NS
Laboratory values			
Albumin	29.7 ± 0.8	29.1 ± 1.8	NS
HbA1c	8.9 ± 0.2	8.4 ± 0.4	NS
Side			
Right	58 (52.7)	15 (50.0)	NS
Left	52 (47.3)	15 (50.0)	
Vascular surgeon	66 (60.0)	16 (53.3)	NS
LOS (mean ± SE, days)	6.3 ± 0.5	8.6 ± 1.0	0.035
Death	33 (32.7)	10 (37.0)	NS

*SE* Standard error, *HbA1c* Haemoglobin A1c, *BKA* Below-knee amputation, *AKA* Above-knee amputation, *LOS* Length of stay, *NS* Not significant

*Statistically significant difference

^↑^Significantly greater than expected (*p* < 0.05)

^↓^Significantly less than expected (*p* < 0.05)

### Annual distribution

The annual distribution of MLEA patients showed a significant increase in the number of surgeries performed by a vascular surgeon over the past 3 years (2015 to 2017) (*P* < 0.05). This correlated with a significant decrease in the revision rate during the same period (*P* < 0.05) (Table 3). Finally, the LOS was significantly longer in 2012 than in subsequent years (*P* < 0.01) (Table 3).

**Table 3:** Annual distribution of major lower limb amputations in northern Jordan between 2012 and 2017

Variable	2012	2013	2014	2015	2016	2017
Frequency (%)	14 (10.0)	31 (22.2)	28 (20.0)	30 (21.4)	17 (12.1)	20 (14.3)
Level of amputation, n (%)						
BKA	11 (78.6)	27 (87.1)	23 (82.1)	21 (70.0)	11 (64.7)	17 (85.0)
AKA	3 (21.4)	4 (12.9)	5 (17.9)	9 (30.0)	6 (35.3)	3 (15.0)
Vascular surgeon, n (%)	3 (21.4)^↓↓^	13 (41.9)	16 (57.1)	20 (66.7)	12 (70.6)	18 (90.0)^↑↑^
Revision, n (%)	4 (28.6) ^↑^	2 (6.5)	6 (21.4) ^↑^	0 (0.0)^↓^	1 (5.9)	1 (5.0)
LOS (mean ± SE, days)	11.9 ± 2.0^↑↑^	6.2 ± 0.9	60.0 ± 0.9	5.2 ± 0.5	6.5 ± 0.9	8.0 ± 1.5

*BKA* Below-knee amputation, *AKA* Above-knee amputation, *LOS* Length of stay, *n* Number, *SE* Standard error of the mean

^↑^Significantly greater than expected (*p* < 0.05)

^↑↑^Significantly greater than expected (*p* < 0.01)

^↓^Significantly less than expected (*p* < 0.05)

^↓↓^Significantly less than expected (*p* < 0.01)

### Length of hospital stay

The LOS was significantly associated with the level of amputation. Patients who underwent AKA had a significantly (*P* = 0.035) longer LOS than those who underwent BKA (Table 2). The mean LOS was 6.3 days for BKA and 8.6 days for AKA (*P* = 0.035) (Table 2). However, there was no significant association between LOS and any comorbidity or any type of aetiologies. The LOS improved significantly over the period of study (from 11.9 days in 2012 to 8.0 days in 2017, *P* = 0.003).

### Mortality

The mortality rate within 1 year of amputation was significantly associated with patient age, female sex, different types of comorbidities, and the preoperative albumin level. Patients who died within 1 year of amputation were significantly older than surviving patients (*P* < 0.001) (Table 4). In addition, a significantly greater number of patients who died had hypertension (*P <* 0.001), stroke (*P =* 0.005), and CKD (*P* = 0.049). In fact, it is important to note that 100% of the amputee patients who died with 1 year had hypertension. However, significantly (*P =* 0.023) fewer than expected amputee patients who died within 1 year had Buerger disease (Table 4). Moreover, patients who died within 1 year of amputation had significantly lower serum albumin levels than other amputee patients (*P =* 0.023). Furthermore, males had a significantly (*P* = 0.008) lower mortality rate. After controlling for all factors in the model, the logistic regression analysis confirmed that the level of amputation, age and stroke were the main factors that significantly increased the risk of 1-year mortality. Finally, there was no significant association between 1-year mortality rates and indications for amputation (Table 4).

**Table 4:** Distribution and analysis of preoperative and postoperative factors related to mortality within 1 year after major lower limb amputation in northern Jordan

Preoperative and postoperative variables	Mortality at 1 year		*P*-value
	Yes N (% from total mortality)	No N (% from total survival)	
Sex			
Male	19 (44.2)	58 (68.2)	0.008*
Female	24 (55.8)	27 (31.8)	
Age (mean ± SE, years)	68.9 ± 1.5	60.1 ± 1.4	< 0.001*
Comorbidities			
Hypertension	43 (100.0)^↑↑^	60 (73.2)	< 0.001*
Diabetes mellitus	40 (87.1)	74 (93.0)	NS
Ischaemic heart disease	21 (50.0)	40 (48.2)	NS
Congestive heart failure	16 (39.0)	25 (30.1)	NS
Stroke	18 (42.9)^↑↑^	16 (19.0)	0.005*
Chronic kidney disease	16 (38.1)^↑^	19 (22.4)	0.049*
Dyslipidaemia	28 (65.1)	56 (65.9)	NS
Buerger’s disease	2 (4.7)^↓^	15 (19.0)	0.023*
Indication for amputation			
Acute limb ischaemia	5 (11.6)	11 (12.6)	NS
Chronic limb ischaemia	3 (7.0)	17 (20.0)	
Diabetic foot syndrome	35 (81.4)	57 (67.1)	
Ulceration	39 (90.7)	70 (82.4)	NS
Laboratory values			
Albumin	27.2 ± 1.1	30.6 ± 1.0	0.023*
HbA1c	8.6 ± 0.4	9.0 ± 0.3	NS
Level of amputation			
BKA	33 (76.7)	68 (80.0)	NS
AKA	10 (23.3)	17 (20.0)	
Side			
Right	25 (58.1)	42 (49.4)	NS
Left	18 (41.9)	43 (50.6)	
Bilateral amputation	14 (32.6)	22 (25.9)	NS
Revision	2 (4.7)	11 (12.9)	NS
Vascular surgeon	21 (48.8)	51 (60.0)	NS
LOS (mean ± SE, days)	6.1 ± 0.6	7.4 ± 0.7	NS

*SE* Standard error, *HbA1c* Haemoglobin A1c, *BKA* Below-knee amputation, *AKA* Above-knee amputation, *LOS* Length of stay, *NS* Not significant

*Statistically significant difference

^↑^Significantly greater than expected (*p* < 0.05)

^↑↑^Significantly greater than expected (*p* < 0.001)

^↓^Significantly less than expected (*p* < 0.05)

## Discussion

In developing countries, the impact of MLEA is increased by poverty, lack of medical insurance, and cost containment policies in deprived nations [13]. Furthermore, there is a wide variation in the reported indications for MLEA. Unlike developed countries, where peripheral arterial disease remains the most common indication for MLEA [14–17], in our series, DFS was the leading indication for MLEA, and this was consistent with other reports from developing nations [18–22]. This is not surprising given that diabetes leads to peripheral arterial disease and three-quarters of MLEA procedures are performed for diabetic patients [11]. Furthermore, diabetes has become an epidemic health problem, with a 32% increase internationally and a 31.5% increase nationally over the past decade [9, 23]. The prevalence of diabetes in Jordan has been estimated to be 17.1%, and the majority of the diabetic population has poor control over their condition [23]. The incidence of diabetic foot ulcer (DFU) reported by the national centre for diabetes in Jordan is 4.6% [24]; however, it is 2% in most developed countries [13, 25, 26]. Moreover, the majority of diabetic patients undergoing MLEA have DFU as a predisposing factor; therefore, MLEA is a potentially preventable public health disease.

The male predominance of 61.4% in our sample is consistent with other reports [8, 9, 11, 15, 16, 25, 27–29]. The mean age of the population in this study was 62.9 years, which is relatively younger than that of other study populations in the literature [8–11, 15, 17, 27, 30, 31]. However, it is comparable with that presented in studies performed in developing countries [18, 19, 22, 32]. This observation can be explained by the fact that diabetes is known to increase the risk of amputation at a younger age, especially when it is acquired at a young age and the vast majority of our patients had diabetes and its related complications [11, 12, 31, 33]. Not surprisingly, the prevalence of comorbid conditions was extremely high in the studied population. More than three-quarters of our patients had diabetes, which represented a higher percentage than that in other contemporary series regarding MLEA [15, 16].

The prevalence of comorbidities was not statistically different in the AKA and BKA groups; however, the BKA group had more patients with CKD than the AKA group (32.1% vs. 14.3%; *P* = 0.047), and this observation was consistent with published data [30, 31, 34]. The BKA:AKA ratio (3:1) in our series was significantly higher than that reported in the literature, including ratios reported by large international registries in developed countries [10, 34]. Recent data from the British National Vascular Registry reported that approximately two-thirds of major lower limb amputations performed between 2014 and 2016 were BKA, and approximately one-third were AKA [35]. We assume that the ratio observed in our study reflected delayed referrals and inadequate assessments during early stages. Therefore, many of our patients presented with an adverse event that rendered limb salvage attempts impossible or ineffective. The EuroDIALE study emphasised the issue of delayed referrals and only 27% of patients who had DFU for 3 months or more were referred for specialty care. Furthermore, only 40% of those with peripheral arterial disease were referred for vascular assessment or revascularisation [36]. In addition, DFS, with its distinct crural vessel involvement and rapidly progressive clinical course, might well explain the high proportion of BKA when DFS accounted for nearly 72.9% of the cumulative MLEA procedures in our series.

The ipsilateral revision rate of BKA to AKA in our study 10.0%) was in accordance with revision rates reported by *Aulivola* et al. and *Dillingham* et al. [7, 12] However, our revision rates were lower than those reported by *Cruz* et al. (12%) and *Lim* et al. (17.6%) [10, 15], and higher than those reported by *Unnikrishnan* et al. [18] The immense disparity in the revision rates reported in the literature may be due to poor primary assessment of the amputation level by training residents, poor management of the amputation stump, and the high risk of falls for this frail patient cohort, all of which may lead to stump dehiscence. Our favourable revision rates might be explained by the increased number of surgeries performed by vascular surgeons over the past 6 years, with 90.0% of MLEA procedures performed by vascular surgeons in 2017 compared to 21.4% in 2012. This corresponded with a significant decrease in revision rates from 28.6% in 2012 to 5.0% in 2017. A recent study in Australia noticed a similar trend in MLEA performed by vascular consultants in terms of complication rates, revision rates, and LOS [16]. This observation supported previous published data regarding the importance of clinical judgement. Although objective data regarding various clinical parameters such as transcutaneous oxygen and segmental Doppler pressure aid are clinically available, these tests cannot replace clinical experience and expertise [12, 15, 16].

Many studies have investigated the outcomes of procedures involving resident-level physicians. *Lannuzzi* et al. studied this issue and reported no significant association between resident involvement and mortality; however, the amputation failure rate was increased [37]. In addition*, Smith* et al. reported that more revision surgeries were required when surgeries were performed by residents instead of consultants [38]. However, *Campbell* et al. found no relationship between the skill level of the operating surgeon and outcomes in terms of local complications and the need for revision [39]. In our study, the type of operating surgeon did not influence mortality and similar mortality rates were observed for vascular and non-vascular surgeons. We did not explore the influence of the skill level of the operating surgeon. In 2016, The Vascular Society of Great Britain and Ireland Amputation Quality Improvement Framework recommended that MLEA should be performed using a planned operating list in the presence of a consultant vascular surgeon [40]. Another interesting finding was that nearly one-quarter of the studied population underwent contralateral amputation following the index MLEA during the study period.

There is no doubt that MLEA is associated with a dismal survival rate [12, 29]. British and American reports have revealed high 1-year mortality rates for MLEA (between 35.7 and 48.3%) [16]. The 30.7% 1-year mortality rate in our study was similar to that reported by other contemporary investigators [7, 15–17, 29, 34]. Our data suggests that female gender, comorbidities such as hypertension, CKD, history of stroke, malnutrition in the form of hypoalbuminaemia, and older patient age were independent factors that increased the risk of 1-year mortality. The logistic regression analysis, after controlling for all factors in the model, confirmed that the main factors that significantly increased the risk of 1-year mortality were the level of amputation, older age and stroke. Previous studies also demonstrated the negative impact of the aforementioned factors on mortality [9, 16, 30].

Other factors, including indication for amputation, history of cardiac disease, and diabetes, did not predict increased 1-year mortality in our patient cohort. Similarly, glycated haemoglobin (HbA1c) did not predict increased mortality following MLEA. Studies have reported increased long-term mortality rates for diabetic individuals who have undergone MLEA; however, reports suggest that there were no significant differences in 1-year mortality rates between diabetic and non-diabetic patients who have undergone MLEA [41, 42]. *Schofield* et al. only noticed increased mortality rates after MLEA for diabetic patients 4 years after amputation, and the 1-year mortality rate was similar to that of non-diabetic amputees, which was in agreement with our results at 1 year. Other investigators have reported conflicting data that indicated survival benefits for diabetic patients [43]. Some reports indicated that preoperative hypoalbuminemia is a marker of adverse outcomes following MLEA [8, 9, 27, 30]. *Feinglass* et al. found that a low serum albumin level was the only preoperative laboratory value predictive of 30-day mortality [27]. *Nelson* et al. also linked increased mortality to hypoalbuminaemia. Interestingly, they did not include it as a parameter in their mortality prediction model [30]. The only modifiable variable associated with significant protection from mortality was Buerger disease. This was probably because this disease is not associated with cardiovascular risk factors, and it rarely involves coronary and cerebral circulation. Furthermore, higher albumin levels in male patients (31.1 for males versus 27.4 for females; *P* = 0,01) and increased occurrence of Buerger disease among males (*P* = 0.018) might explain favourable mortality rates in males.

Despite the recent advances in perioperative care and endovascular tools, the mortality rates for MLEA in our study were not significantly better. This might be due to selection bias that can arise because patients who are offered amputation are naturally sicker than those undergoing revascularisation, and they have worse health preoperatively than patients scheduled to undergo a limb salvage procedure [7, 15, 27, 31, 44].

In the current era of advanced endovascular therapy, minimally invasive revascularisation might be associated with more favourable outcomes than MLEA [30, 44]. Moreover, referrals to tertiary care units in our country are subject to the annual funds allocated by the Ministry of Health, which vary on an annual basis and are dependent on the overall allocated annual allowances for such units. Therefore, the number of referrals varies on a yearly basis, and sicker patients take priority when referrals to our unit are made.

Declining amputation rates have been reported in developing countries [14, 17, 34]. In addition, our data did not reveal any significant change in the annual amputation rates despite improved vascular consultant availability and the introduction of advanced vascular and endovascular tools at our institution. Furthermore, we did not notice any consistent change in the level of amputation over the years since 2012. This might indicate that limb salvage options are not effective, or that they are not offered early in developing countries such as Jordan. The development of primary care prevention strategies is crucial for prevention and early management of foot ulcerations, which, according to our data, are present in 84.3% of patients prior to MLEA.

We noticed a 4.2-fold increase in vascular surgeon involvement in MLEA procedures over the past 6 years. Vascular surgeon-led MLEA was also associated with a corresponding decrease in amputation revision rates from 28.6% in 2012 to 5.0% in 2017, and a shorter LOS from 11.9 days in 2012 to 8.0 days in 2017; presumably resulting in decreased overall costs of MLEA. However, further nationwide studies are required to confirm that shorter LOS, reduced revision rates, and vascular surgeon-led procedures actually result in reduced costs.

LOS remains an important determinant of health service effectiveness, overall cost, patient wellbeing, and patient satisfaction [19]. In our study, the mean acute postoperative LOS was 6.8 ± 0.4 days, which was similar to that reported by *Wise* et al. [28] LOS was significantly affected by the level of amputation, with BKA having a significantly shorter LOS than AKA (6.3 vs. 8.6 days; *P =* 0.028). Our overall LOS was shorter than that mentioned in contemporary reports from developed countries, which can be explained by the absence of rehabilitation services at our hospital. Patients at our hospital are discharged home when pain is controlled, and the wound is healing.

We were not able to demonstrate any significant influence of sex, select comorbidities, or laboratory values. Interestingly, we noticed a counterintuitive direct relationship between hospital LOS and 1-year survival, which might be explained by the fact that we included in-hospital deaths in the 1-year mortality rate; however, this did not reach statistical significance. That is, a substantial number of patients died during the perioperative period and, therefore, were considered discharged earlier, as per hospital records. Establishing a national vascular registry is crucial so that we can establish factors associated with shorter LOS. This will assist in providing a cost-effective service that maintains quality care while reducing expenses.

Several authors have explored the importance of developing a foot care team to decrease the risk of MLEA [14, 26, 45, 46]. *Rogers* et al. discussed this issue in their comprehensive review and introduced the Toe and Flow concept, which is a comprehensive clinical approach to endangered limbs. Furthermore, *Fitzgerald* et al. introduced the diabetic rapid response (DRRAFT) concept model, which involves seven essential skills that form the necessary core of the interdisciplinary limb salvage model [46]. *Paisey* et al. confirmed that major cost-savings resulted from reduced diabetes-related major limb amputations with a 75% decrease in the amputation cost per person per year when a foot care service was implemented [45].

In our opinion, implementation of a foot care pathway should not be restricted to diabetic foot patients. It should also be extended to those with critically ischemic limbs. Investigators from the United Kingdom highlighted this issue when they noticed a slower decrease in amputation rates for non-diabetic patients with ischaemic limbs than for diabetic patients, and therefore, they recommended that campaigns such as “putting feet first” should not be restricted to diabetic patients with critical limbs [14].

This study had some limitations. First, our study was retrospective and involved a relatively small sample size with less than 30 MLEA per annum. Second, there were gaps in the database, and there may have been potential selection bias. Third, this was a single-centre study, and the patient population encountered in this tertiary centre may have had more complex and advanced comorbidities. Therefore, they might not represent the whole population. Fourth, data regarding different variables that impact outcomes of MLEA, such as socioeconomic status, functional status, and other laboratory variables, were lacking. Fifth, diabetes mellitus and peripheral arterial disease frequently occur together, and therefore, precise classification is often impossible. Finally, data regarding long-term outcomes, such as patient ambulation and quality of life, were lacking.

## Conclusion

In conclusion, Vascular surgeon-led MLEA is associated with decreased revision rates, LOS and possibly improved outcomes, particularly when it is performed for vascular insufficiency. The incidence of MLEA is significantly and inversely correlated with the provision of foot care services [45]. Therefore, imminent research exploring factors associated with increased costs for MLEA is crucial for formulating health care polices in developing countries. We hope that this study will provide a platform for implementation of new health initiatives that bring about improvements in patient outcomes in developing nations. We also hope that our findings encourage health care policymakers to support health care professionals in their undertaking of national foot awareness campaigns. Finally, the introduction of a national vascular registry is imperative for exploring regional and patient-specific variations in outcomes following MLEA in Middle Eastern and North African countries.

## Data Availability

The datasets generated and analyzed during the current study are available from the corresponding author.

## Abbreviations

AKA: Above-knee amputation
ANOVA: Analysis of variance
BKA: Below-knee amputation
CKD: Chronic kidney disease
DFS: Diabetic foot syndrome
DFU: Diabetic foot ulcer
IRB: Institutional review board
KAUH: King Abdullah University Hospital
LOS: Length of hospital stay
MLEA: Major lower extremity amputation
SE: Standard error
